# Service based comparison of group cognitive behavior therapy to waiting list control for chronic fatigue syndrome with regard to symptom reduction and positive psychological dimensions

**DOI:** 10.1097/MD.0000000000016720

**Published:** 2019-09-27

**Authors:** Adrian Heald, Louise Barber, Helen Lyon Jones, Sanam Farman, Andreas Walther

**Affiliations:** a Department of Endocrinology; b Department of Clinical Psychology, Salford Royal NHS Foundation Trust, University of Manchester, Salford; c Mersey Deanery Psychiatry Rotation, Liverpool, UK; d Biological Psychology, TU Dresden, Dresden, Germany.

**Keywords:** chronic fatigue syndrome, depression, group cognitive behavior therapy, hope, optimism

## Abstract

**Background:**

Although chronic fatigue syndrome (CFS) sometimes referred to as myalgic encephalomyelitis (ME) is a very challenging condition to treat, there is evidence that individual cognitive behavioral therapy (ICBT) can be effective for treatment and management of its symptoms. Furthermore, group cognitive behavioral therapy (GCBT) is emerging as promising treatment for the condition.

The aim of the present study was to explore further the effectiveness of GCBT in a routine clinical setting and to investigate associated positive psychological effects related to GCBT.

**Methods:**

In this pragmatic, non-randomized, controlled trial, 28 people acted as their own waiting list control by completing a range of measures 8 weeks prior to taking part in the GCBT. The intervention consisted of 8 consecutive weeks of 2.5-hour sessions.

**Results:**

Repeated measures analysis of covariance revealed significant improvements in physical fatigue (*F* = 28.31, *P* < .01, effect size *d* = 0.52), mental fatigue (*F* = 7.72, *P* < .01, effect size *d* = 0.22), and depressive symptoms (Beck depression inventory-fast screen for medical individuals [BDI-FS]: *F* = 11.43, *P* < .01, effect size *d* = 0.30; hospital anxiety and depression scale [HADS-D]: *F* = 16.72, *P* < .01, effect size *d* = 0.38) compared with the waiting list. Improvements in quality of life (*F* = 7.56, *P* < .01, effect size *d* = 0.23), hope (*F* = 15.15, *P* < .01, effect size *d* = 0.36), and optimism (*F* = 8.17, *P* < .01, effect size *d* = 0.23) were also identified, but no change was reported for anxiety levels. Global outcome measures revealed that the majority of the individuals found the treatment beneficial and were satisfied with the results.

**Conclusion:**

GCBT is a beneficial and cost-effective treatment that individuals find amenable in routine clinical practice for CFS. Additionally we have described important effects emerged on positive psychological dimensions such as hope and optimism potentially enhancing the overall benefit.

## 1 Introduction

Chronic fatigue syndrome (CFS) sometimes referred to as myalgic encephalomyelitis (ME)^[1]^ is a very challenging condition to treat. CFS consists of severe fatigue lasting at least 6 months accompanied by a range of other symptoms including sleep disturbance, difficulties with concentration and memory, headaches, and musculoskeletal discomfort.^[2–4]^ Individuals with CFS attend a variety of speciality clinics such as Rheumatology, Endocrinology, Infectious Diseases, and Psychiatry depending on availability of specialist expertise at a local and regional level.

Although as yet the cause is unknown, there are a range of different interventions used in the treatment and management of CFS. United Kingdom (UK) National Institute for Health and Care Excellence (NICE) guidelines are currently under review and have previously recommended Cognitive Behavioral Therapy (CBT) as the most effective therapeutic intervention for people with mild to moderate CFS, along with graded exercise therapy (GET).^[5–7]^ However individual response is very variable.

Group therapy is becoming increasingly popular as a cost-effective intervention in clinical services that are publicly funded.^[7]^ It may be useful for reducing time spent on a waiting list and can be particularly advantageous where an intervention has a significant psycho-educational component.^[8]^ Group therapy also enables a peer support element that is not available in individual therapy but can have therapeutic benefits to CFS sufferers, who often face misunderstanding and disbelief from non-sufferers.^[9]^ These positive aspects of group therapy might elicit further positive psychological effects such as improving optimism, hope, or quality of life.

To date, there have been a small number group CBT (GCBT) trials. Saxty and Hansen's^[8]^ preliminary study of GCBT efficacy in CSF individuals reported that most individuals showed improvements for fatigue, general health, and social adjustment on pre- and post-group scores. However, the study was limited by a small sample of 6, all of which were women and so it is difficult to generalize from the findings. The researchers themselves concluded that a controlled trial was required to ascertain if GCBT may be more beneficial.

Bazelmans et al^[10]^ used a larger sample size in their non-randomized, waiting list control design to study the effectiveness of CBT on a group of unselected individuals with CFS in 2 centers in the Netherlands. An insignificant interaction effect was identified in favor of GCBT for fatigue. Functional impairment did not change in people in the GCBT arm but declined in the waiting list control.

O’Dowd et al^[11]^ subsequently undertook a double-blind, randomized, controlled trial compared 3 interventions; group cognitive behavioral therapy (GCBT), education with support group (EAS), and standard medical care (StMC). Follow-up data was collected at 6 and 12 months. Of the 153 participants, two-thirds were women. They reported no significant change in cognitive function or quality of life as a result of participation in the GCBT group but the GCBT group reported less fatigue, and improved walking speed compared with those in the StMC group. These improvements persisted when compared with those in the EAS group.

A more recent study in 2015 undertaken by Wiborg et al^[12]^ compared 136 CSF individuals receiving GCBT and 68 CSF individuals in the waiting list control group. It revealed a large and significant positive effect in favor of the intervention group on fatigue severity and overall impairment.^[12]^ Physical functioning and psychological distress also improved moderately in this study. However there were factors which should be taken into account, notably that the GCBT was delivered by highly motivated therapists in an expert center for CFS, which might partially account for the large effect sizes. Second, only participants who were willing to receive group therapy were included in the trial and those participants preferring ICBT, were allocated to the waiting list. This potentially confounded the participants’ motivation by increasing it for the GCBT and reducing it for the ICBT individuals.

The aim of the study that we report in this paper, was to investigate the effectiveness of GCBT for CFS in a routine clinical setting within a UK public health setting, addressing concerns regarding ecological validity and generalization of findings to other specialist CFS services. Our study appears to be unique in so far as it is the only study that has explored hope and optimism as potential outcome variables in people with CFS. These variables were reviewed by Schiavon et al^[13]^ in 2016. They described that there is a close relationship between the constructs of optimism and hope and a reduction in the effects of chronic disorders.

The hypothesis was that an 8-week GCBT intervention would be superior to an 8-week waiting period on measures of quality of life, mental health and fatigue, as well as positive psychological measures such as optimism and hope. We present our findings here.

## 2 Aim of the study

As little research has been conducted to explore the effectiveness of GCBT for CFS, this study aimed to use a repeated measure waiting list control to examine whether GCBT is an effective treatment in routine clinical practice. The hypothesis was that individuals would show changes presenting less CFS symptomatology and better scores in the optimism and hope scales after the 8-week GCBT compared with an 8-week waiting list control.

## 3 Methods

**Figure 1:**
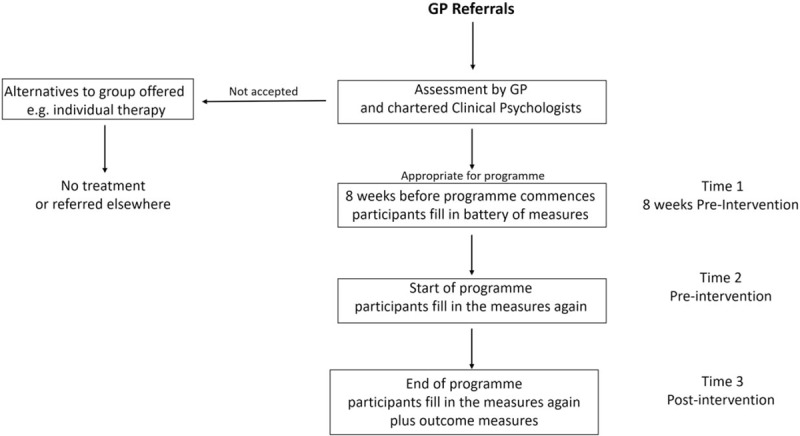
The study procedure/design.

Participants were screened and tested over a 2-year period (Fig. 1: the study procedure/design). Participants were consecutive general practioner (GP) referrals to a specialist clinic in North Wales, UK. Individuals were eligible to take part in the study if they were over 18 years old and met the Oxford criteria for CFS.^[14]^ This specifies the presence of medically unexplained, new onset disabling fatigue of at least 6 months duration and mental, as well as, physical fatigue. All participants had also been deemed amenable and appropriate for group work.

Using Cohen's^[15]^ power primer a calculation was made. With the significance criteria set at 0.05, the power calculation deemed a necessary sample size to be 34. This number was not achieved due to a lower rate of referrals than was expected. Of the 30 individuals assessed as suitable for a group programme, 2 subsequently withdrew from treatment before the start of the intervention for unspecified reasons. Three groups of 8 to 10 individuals were completed. Of the 28 participants, who took part in the study, 35.7% were taking antidepressants at assessment and throughout treatment. All the individuals classed themselves as white British (English/Welsh). Further demographics are outlined below in Table 1.

**Table 1:**
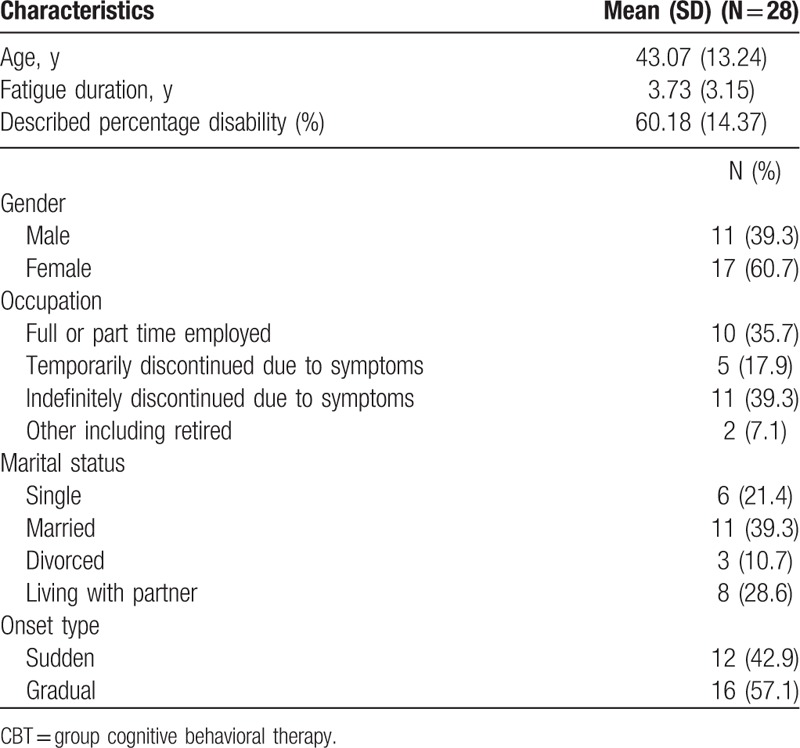
Demographics of people receiving CBT.

Approval for the study was obtained from the Local Research Ethics Committee to monitor the effectiveness of group CBT for adult CFS sufferers in an NHS service in North Wales. Participants gave written informed consent for their data to be used in the present study.

In line with Quarmby et al's^[16]^ concerns regarding the ecological validity of randomized control trials (that the process of patient selection in a randomized control trial may lead to differences between the groups), it was decided to devise a novel repeated measures design, where individuals act as their own control. This resulted in a waiting list control group that was “service friendly” in that the waiting period to treatment was not artificially increased, service level agreements were not compromised, and comorbid individuals or those on medication were not excluded from the protocol.

Screening and Exclusion Criteria: Individuals were selected as suitable for group therapy on the basis of joint assessment by a Consultant Physician with expertise in CFS and a Clinical Psychologist with experience in CFS. Patients were excluded if they took anti-depressant or anxiolytic medication of >10 mg/d/diazepam or equivalent, or if their dose changed during the trial or within the 3 months prior. Patients with somatisation disorder, severe depression, ongoing physical investigations, concurrent treatment, and/or an inability to attend all therapy sessions were also excluded. This is in line with previous studies.

As in routine clinical practice, participants completed a battery of questionnaires at the start and end of the 8-week intervention. For the purposes of the study the battery was extended to include participant outcome measures and positive psychological measures. Similarly, at the end of the programme the views of the individuals “significant others” were assessed. They were asked to consent in writing to provide their perception of the outcome of the intervention.

## 4 Psychometric measures

Having collected the participants’ basic demographic and employment details the following measures were used:

### 4.1 Fatigue questionnaire

This 11-item fatigue questionnaire asks individuals to rate their fatigue on a 4-point scale from “less than usual” to “much more than usual.”

### 4.2 Beck depression inventory-fast screen for medical individuals (BDI-FS)

The BDI-FS^[17]^ consists of 7 items derived from the larger BDI-II.^[18]^ This instrument was designed to assess the intensity of depression in terms of 21 symptom-attitude categories. The Fast Screen was developed to assess depression specifically in individuals reporting with comorbid somatic and behavioral symptoms, otherwise attributable to biological, medical, or substance abuse problems.

### 4.3 Hospital anxiety and depression scale (HADS)

The HADS^[19]^ is a 14-item scale with 2 7-item sub-scales measuring depression and anxiety. Like the BDI-FS each statement is ranked for symptom severity from 1 to 4. The HADS is extensively used in clinical settings and has good validity.

### 4.4 Quality of life visual analogue scale as part of the EUROQOL (EQ-5D)

The EQ-5D is a standardized measure of health outcome.^[20]^ Applicable to a wide range of health conditions and treatments, it provides a simple descriptive profile and a single index value of health status. Only the visual analogue scale of the EQ-5D was used in this study.

### 4.5 Life orientation test (LOT)

This 10-item measure of dispositional optimism includes 4 filler items, 3 positively worded items, and 3 reversely coded items. Participants rated each item on a 5-point Likert scale ranging from 1 (strongly disagree) to 5 (strongly agree). Where higher scores correspond with increasing of optimism. This has been shown to have adequate reliability, predictive validity, and discriminate validity.^[21]^

### 4.6 State hope scale

The adult state hope scale was developed from a dispositional hope scale and consists of 6 hope items, which are designed to measure goal directed cognitions.^[22,23]^ It is comprised of 2 sub-scales. The “agency” sub-scale is made up of 3 items, which measures the perceived motivation to move towards goals. The “pathways” sub-scale is made up of 3 items, which measure the perceived ability to generate workable routes to goals.

### 4.7 Global self-ratings and rating by other informant (following the intervention)

Overall improvement, fatigue, and disability were measured on 6-point scales from “very much better” to “very much worse.” Similarly, satisfaction with treatment on a 7-point scale from “very satisfied” to “very dissatisfied,” and usefulness of treatment on a 5-point scale from “very useful” to “no use at all” was measured.

## 5 Intervention

The therapy consisted of 8 sessions, over an 8-week period and was facilitated by a clinical psychologist, GP, dietician, and a physiotherapist, all of whom had at least 8 years experience of GCBT for CFS sufferers. Each session lasted 2 and a half hours with a 15-minute break, for refreshments and a chat. All the therapists adopted a positive, informal, and friendly approach. Throughout the programme, members of the group were encouraged to focus on solutions and reward themselves with regular treats. Conversely, they were discouraged from excessive symptom focus. Regular time for joke telling was scheduled to further facilitate a non-adversarial atmosphere and increase acceptability of the intervention. Links between sense of humor and self-reported physical health appear to be well supported.^[24]^ Similarly, there is a growing body of empirical data to support the popular belief that laughter benefits health (Fig. 1).^[25]^

## 6 Ethics approval and consent to participate

Ethics committee approval was obtained and all participants gave fully informed consent.

## 7 Statistical analysis

All analyses were performed using SPSS version 23.0.0 for MAC OSX 10.12.6 (Armonk, NY: IBM Corp.). The data were checked for normality using a one sample Kolmogorov-Smirnov test of goodness-of-fit for all the computed variables (*Z* ≤ 0.579, *P* ≥ .242). All variables in the analysis demonstrated a parametric distribution and were suitable for parametric analysis.

Repeated measures analysis of covariance (ANCOVA) was used for identifying statistical differences between some or all of the means of the groups by comparing them to the grand mean. Bonferroni adjusted pairwise comparisons were used to look for differences between the time points as there is some doubt whether the Tukey test affords sufficient protection from inflation type one errors.^[26]^ Adjustments were made for the multiple comparisons. All participants completed all the time point measures, although one participant refused to fill in one of the measures as he “disagreed with its theoretical assumptions,” hence there is very little missing data.

## 8 Results

All individuals attended at least 5 of the 8 sessions and more than half the individuals attended all 8 sessions. Segal et al,^[27]^ report 4 sessions to be a minimum requirement to be able to evaluate treatment effectiveness. The data file was split and *t* tests were used to look for differences between those who attended all 8 sessions and those who didn’t. No significant differences were found for all the baseline measures between the waiting list individuals and participants. Specifically participants and waiting list individuals did not differ at the start in terms of their summative scores on the Fatigue questionnaire.

**Figure 2:**
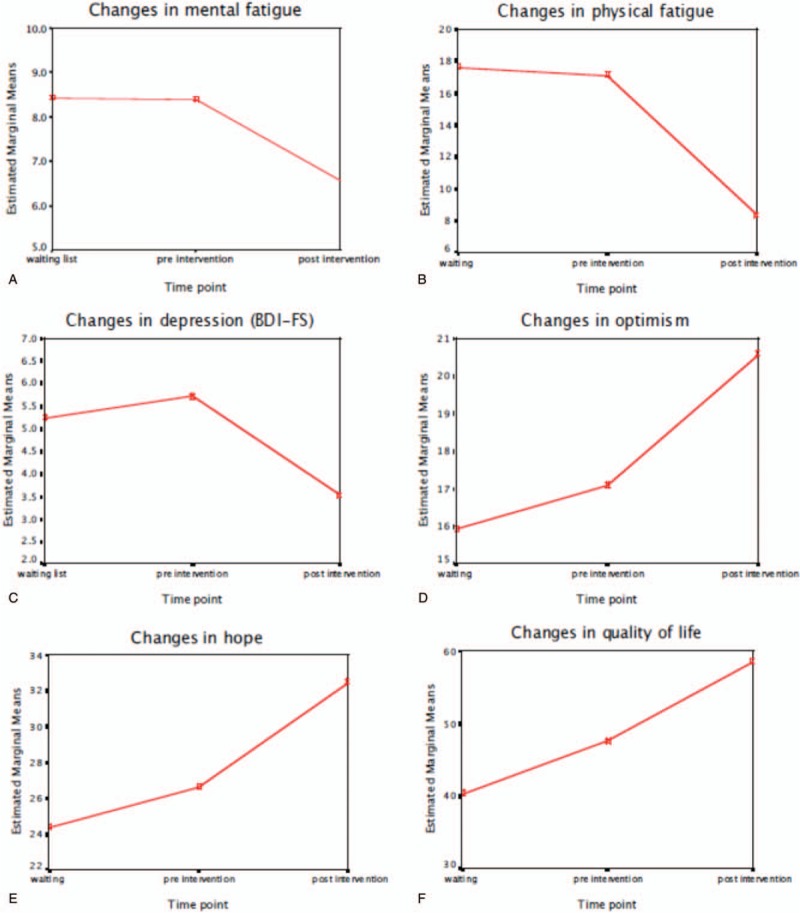
A–F: Changes in principal measures for the waiting list stage and intervention stage.

The overall attendance rate for the 28 individuals was 90.2%. Table 2 and Fig. 2A–F show the results of the repeated measure ANCOVA's.

**Table 2:**
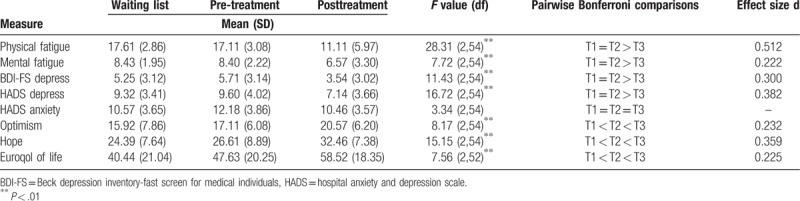
Mean and standard deviation scores for the waiting list control and pre- and post-intervention.

### 8.1 Fatigue outcome

Results of the repeated measures ANCOVA demonstrated significant intervention related reductions in physical and mental fatigue scores. Bonferroni adjusted pairwise comparisons in Table 2 revealed significant difference between the pre and post intervention outcome but not for the waiting list and pre intervention scores (see Table 2). This indicates that individuals fatigue scores improved as a function of the intervention and not as a function of waiting for the intervention. Medium effect sizes were found for both, physical and mental fatigue scores (Fig. 2A and B).

### 8.2 Mood measures

Significant intervention related reductions were found for both psychometric instruments of depression scores. Bonferroni adjusted pairwise comparisons demonstrated a significant difference between the pre- and post-intervention outcome but not the waiting list and pre-intervention scores. This also indicates that the individual's depressive symptoms scores improved as a function of the intervention and not as a function of waiting for the intervention. Significant medium effect sizes were again found for both scores (see Table 2). However, no significant difference was found for HADS Anxiety scores indicating no effect on anxiety levels by the GCBT (Fig. 2C).

### 8.3 Positive psychological measures

Significant intervention related increases were found for both optimism and hope measured by the LOT and the state hope scale, respectively. Bonferroni adjusted pairwise comparisons identified significant difference between the pre- and post-intervention outcome, but not the waiting list and pre-intervention scores indicating individual's optimism and hope scores improved as a function of the intervention and not as a function of waiting for the intervention. Significant medium effect sizes were again found for both scores (see Table 2, Fig. 2D and E).

### 8.4 Quality of life measure

With regard to the quality of life (QOL) measure (Fig. 2F), significant differences were found between the different time points. However, Bonferroni adjusted pairwise comparisons show that the QOL difference was significant between the waiting list and post-intervention scores indicating that individual's quality of life increased in the waiting list control and continued to increase following the intervention and is therefore not attributable to the intervention alone.

With regard to self-reported global outcome measures at the end of treatment. Twenty-four participants (85.7%) classed themselves as better to some degree, while 14.3% (4) rated themselves as “about the same” or “worse” after the intervention. With regard to their fatigue 71.4% (20) participants rated themselves as better to some degree, while 28.6% (8) subjects rated themselves as “the same” or “worse” after the intervention. With regard to their disability/restrictions 64.3% (18) people regarded themselves as better to some degree and 35.7% (10) people were classing themselves as “the same” or “worse.” Twenty-five (89.3%) classed themselves as satisfied to some degree with the outcome of the intervention, with 7.1% (2) “neither satisfied or dissatisfied,” and 3.6% (1) rating themselves as “slightly dissatisfied” with the outcome. Everyone found the intervention useful to some degree with 64.3% (18) finding it “very useful.”

Generally the qualitative feedback was positive. Many people commented on the benefit of “validation” Their process of being listened to, believed and supported by the therapy team and other CFS sufferers starts at the assessment stage and continues throughout the intervention. Some individuals also reported the benefits of the social aspects and the realization that they are not alone. However, limitations of this study mean that a full analysis of the qualitative feedback from the participants is beyond its scope. For various reasons many people did not return their informant global outcome measures. However, the data that was returned is summarized in Tables 3 and 4.

**Table 3:**
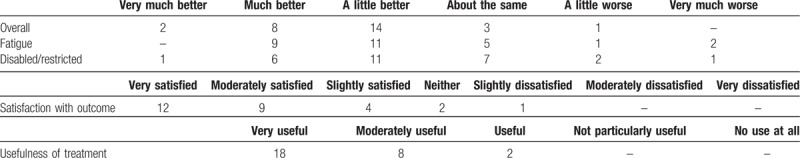
Global outcome scores at the end of treatment (n = 28).

**Table 4:**
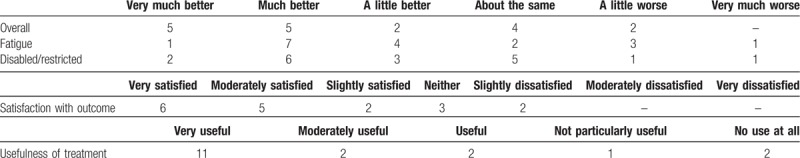
Informant global outcome scores at the end of treatment (n = 18).

## 9 Discussion

Our results further support prior studies showing that GCBT is effective in people with CFS. Positive effects on both mental and physical fatigue were found compared with the waiting list control that showed no significant changes. This supports the preliminary findings on GCBT in CFS of Saxty and Hansen^[8]^ and Wittkowski et al,^[28]^ who reported improvements in self-reported fatigue in their small sample. It is also in line with Nunez et al^[29]^ who reported improvements in physical function and pain following the intervention compared with treatment as usual. It also supports findings from White et al^[7]^ and O’Dowd et al^[11]^ who suggested that GCBT helped to reduce both mental and physical fatigue.

This study appears to be unique in so far as it is the only study that has explored hope and optimism as potential outcome variables in people with CFS. This was reviewed by Schiavon et al^[13]^ in 2016. They described that there is a close relationship between the constructs of optimism and hope and a reduction in the effects of chronic disease. The authors in that paper pointed out that it is important to highlight that the association between optimism/hope and physical health differs does depend on the context of the disease and the subjects.

Furthermore, since the study by Wiborg et al^[12]^ showed in a large sample the effectiveness of GCBT irrespective of group size, the here presented study offers more ecological validity and achieves to replicate these findings in a smaller sample. Furthermore, positive psychological measures such as hope and optimism were, for the first time, shown to be significantly improved by GCBT indicating a potential therapy enhancing effect of the group setting. Importantly, although the intervention used in this study was significantly shorter in overall duration (8 weeks vs 6 months) and number of sessions (8 sessions of 2.5 hours vs 14 sessions of 2 hours) compared with the study by Wiborg et al,^[12]^ very similar effects with generally lower effect sizes were observed.

With regard to mood, the intervention appeared to be effective at reducing depressive symptoms, but not the waiting control. However anxiety remained fairly stable with no significant difference following intervention. These findings support the previous GCBT findings of reduction in psychological distress^[12]^ but contrasts other studies showing little or no change in depression outcome scores or anxiety. Bazelman et al^[10]^ found no changes in psychological well-being or depression and Wittkowski et al^[28]^ also found depression and anxiety symptoms to remain fairly stable following their interventions.

A potential explanation for these results is that mood measures are, perhaps, not that appropriate as outcome measures in CFS intervention studies. The anticipation or experience of taking part in group therapy, for some people may be a novel and perhaps stressful experience. It is also impossible to control for naturally occurring adverse events that effect mood. However, in their ICBT trial Deale et al^[30]^ also reported similar stability of mood. Alternatively, anxiety was particularly high for this clinical sample and so may have needed additional input. However, the identified reduction in depressive symptoms in the here presented study and in the study by Wiborg et al^[12]^ provides evidence for the positive effect of GCBT in CFS sufferers on the mood dimension.

Although, non-significant improvements between pre- and post-intervention were reported for quality of life, this difference was significant between waiting list and post-intervention. Again, this is in line with the quality of life improvements reported in Taylor^[9]^ who utilized some CBT techniques in their individual and group therapy trial. In the current study the improvements appeared to take place while the participants were on the waiting list and continued to improve following the intervention. This could in part be explained by the anticipatory element of their wait (and hope of better management) and is further supported by the results for hope and optimism, which also showed improvements in the 8-week waiting period before the intervention. There was anecdotal evidence from participants that the assessment process alone is beneficial as it is a validating experience. People can often find it hard to get better if others do not believe they are ill in the first place.

There was an improvement for optimism scores in the waiting list condition and this improvement continued after the intervention. However, only the improvement between pre- and post-intervention reached significance. There was a similar pattern with hope as individuals improved while on the waiting list but this improvement did not reach significance. However, the improvement in hope following the intervention did reach significance. This demonstrates a greater improvement as a result of the intervention.

As stated above, this study appears to be unique in so far as it is the only study that has explored hope and optimism as potential outcome variable with individuals with CFS. It also appears that the outcome measures are effective at demonstrating the changes that occur as a result of taking part in the GCBT intervention. It is recommended that other studies also explore the effectiveness of these measures as potential outcome measures following intervention.

As GCBT aims to help individuals manage their condition and utilizes changes in cognitions to help achieve this, it seems intuitive that positive psychological measures be used in conjunction with the standardized mood, pain, and functioning measures, which are perhaps less sensitive to change.

There is a body of evidence supporting CBT and graded exercise therapy (GET)^[31]^ as an effective treatment of CFS. The Cochrane Review of CBT for CFS in adults^[32]^ consisting of 1043 participants across 15 studies illustrated that when comparing CBT with usual care, the result of mean fatigue scores posttreatment were highly significant in favor of CBT. Another study by Quarmby et al,^[16]^ compared the outcomes of individual CBT (ICBT) in randomized control trial to those in routine practice. The results in the randomized control trial showed superior results compared with those in routine practice. The authors suggest this effect may be due to individual selection, in that the randomized control trials are selective in who they chose for their trials. Similarly, individuals for the RCT may have self-selected where the study has been advertised publicly. Other factors for consideration are that therapists are often involved in CFS research and may be somewhat “evangelical” compared with therapists in routine clinical practice. They may also adhere more strictly to a specific treatment protocol, than their colleagues in routine service. Nevertheless it must be pointed out that a significant number of individuals experience only a minimal alleviation of symptoms with CBT.

One potential limitation to the study is it is a relatively small sample size and so the results should be viewed with some caution. Another limitation of the study is that it does differentiate between different symptoms. Even though information is routinely collected regarding symptom profiles this was not analyzed during the study. Many of the individuals differed in their experience of the condition and how they managed it. It could be that the intervention has different effects for different symptoms or personality types (e.g., the integral focus on pacing helps goal oriented people avoid over activity bursts). Future research that addresses the issue of definition and sub-typing might help address this issue.

Finally, it is important to note that evidence from randomized trials do not guarantee success in routine service practice for everyone.^[33]^ As this trial demonstrates, GCBT does not elicit positive result for everyone and there is still much to be learnt about the symptom control and management of CFS.

The National Institute for Clinical Excellence (NICE) is currently reviewing the whole area of CFS treatment. Alternative treatment strategies based on novel pathophysiological mechanisms such as the Perrin technique^[34]^ are being explored. Qualitative research could be used to try to evaluate why the therapy worked for some and not others—this may be a future direction for CFS research that we and others will pursue.

## 10 Conclusion

GCBT is a cost-effective and beneficial treatment that individuals find amenable in routine clinical practice for CFS. Additionally, important effects emerged on positive psychological dimensions such as hope and optimism potentially enhancing the over-all treatment effect. We hope that this paper will be helpful to everyone working in the field.

## Author contributions

**Conceptualization:** Adrian Heald, Louise Barber, Helen Lyon Jones.

**Data curation:** Louise Barber, Helen Lyon Jones.

**Formal analysis:** Louise Barber, Helen Lyon Jones.

**Investigation:** Louise Barber, Helen Lyon Jones.

**Methodology:** Louise Barber, Helen Lyon Jones.

**Validation:** Louise Barber, Helen Lyon Jones, Sanam Farman, Andreas Walther.

**Visualization:** Adrian Heald, Louise Barber, Helen Lyon Jones, Sanam Farman, Andreas Walther.

**Writing – original draft:** Louise Barber, Helen Lyon Jones.

**Writing – review & editing:** Adrian Heald, Louise Barber, Sanam Farman, Andreas Walther.

Adrian Heald orcid: 0000-0002-9537-4050.

Andreas Walther orcid: 0000-0003-4516-1783.
